# Transmission of Hand, Foot and Mouth Disease and Its Potential Driving Factors in Hong Kong

**DOI:** 10.1038/srep27500

**Published:** 2016-06-07

**Authors:** Bingyi Yang, Eric H. Y. Lau, Peng Wu, Benjamin J. Cowling

**Affiliations:** 1 WHO Collaborating Centre for Infectious Disease Epidemiology and Control, School of Public Health, Li Ka Shing Faculty of Medicine, The University of Hong Kong, Hong Kong Special Administrative Region, Hong Kong, China

## Abstract

Hand-foot-and-mouth disease (HFMD) is a common childhood disease with substantial disease burden in Asia. Mixed results were reported on the associations between HFMD incidence and meteorological factors or school holidays, while limited studies focused on their association on transmissibility. We aimed to measure the transmissibility of HFMD and to examine its potential driving factors in Hong Kong. A likelihood-based procedure was used to estimate time-dependent effective reproduction number (*R*_*t*_) based on weekly number of HFMD-associated hospitalizations from 2010 to 2014. The associations of between-year effects, depletion of susceptibles, absolute humidity and school holidays with *R*_*t*_ were examined using linear regression. *R*_*t*_ usually started increasing between early spring and summer and peaked in April to May at around 1.1–1.2, followed by a slight rebound in autumn. Depletion of susceptibles and between-years effects explained most of the variances (19 and 13% respectively) in *R*_*t*_. We found a negative association between depletion of susceptibles and *R*_*t*_ (coefficients ranged from −0.14 to −0.03 for different years), but the estimated effects of absolute humidity and school holidays were insignificant. Overall, HFMD transmission was moderate in Hong Kong and was mainly associated with depletion of susceptibles. Limited impact was suggested from meteorological factors and school holidays.

Hand-foot-and-mouth disease (HFMD) causes a substantial disease burden in Asian regions including Hong Kong, mainly in children below 5 years of age^1^,^2^,^3^,^4^. Enterovirus 71 (EV71), coxsackievirus A16 (CA16) and coxsackievirus A6 (CA6) are the most common enterovirus serotypes causing HFMD in Hong Kong^2^,^5^. HFMD has a clear seasonal pattern in temperate regions with annual peaks in the summer^1^,^6^, while in tropical and subtropical regions including Hong Kong, multiple peaks may occur within a year^2^,^7^. Inconsistent findings about the associations between meteorological factors, such as temperature and relative humidity, and the incidence of HFMD were reported by different studies^6^,^7^,^8^,^9^,^10^,^11^,^12^,^13^,^14^,^15^.

HFMD is considered to be transmitted mainly through direct contact with contaminated discharges, fluid of blisters or stool from infected persons, or contaminated objects^16^. Attending kindergarten or child-care center and visiting public playground were also reported as risk factors for HFMD^17^,^18^,^19^. HFMD is highly transmissible, with a basic reproduction number (*R*_0_) ranging from 2.5 to 5.5 for different serotypes in Hong Kong and Singapore^20^,^21^. A temperature of 21.1 to 26.7 °C, higher relative humidity and school period were reported to be associated with higher HFMD transmission in mainland China^22^. Other factors, such as host susceptibility and virus-specific transmissibility were also found to play vital roles in transmission of infectious diseases^23^. Currently, there is a lack of evidence about the potential risk factors and their impact on HFMD transmission. This study therefore aims to examine the transmissibility of HFMD and its potential driving factors in Hong Kong, where HFMD shows less obvious seasonality.

## Results

**Figure 1:**
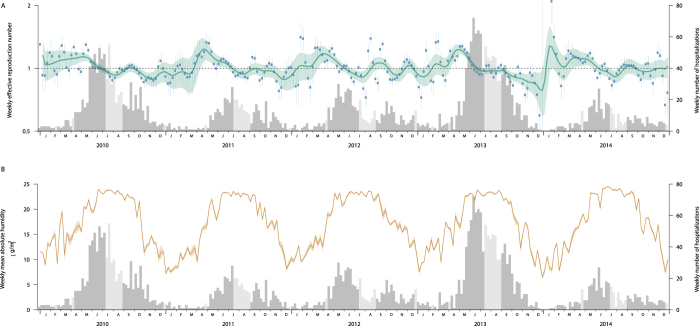
HFMD epidemic and transmission, absolute humidity and school vacations in Hong Kong, 2010–2014. (**A**)Weekly effective reproduction number (*R*_*t*_) of hand foot and mouth disease in Hong Kong, 2010–2014. Blue dots and vertical solid line represent non-smoothed weekly *R*_*t*_ and its 95% confidence interval (CI). Green lines and the green shade represent smoothed weekly *R*_*t*_ and its 95% CI. The dashed line represents the *R*_*t*_ threshold of 1. (**B**)Weekly mean absolute humidity in Hong Kong, 2010–2014. The 95% CI was estimated using bootstrap. The histogram in the two panels represents the weekly number of HFMD-associated hospitalizations while the light grey bars represent weeks in school vacations.

**Figure 2:**
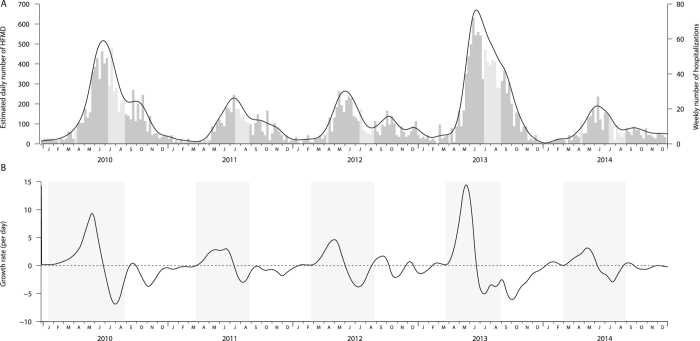
Identification of the main epidemic periods. (**A**)The estimated daily number of hand foot and mouth disease from a smoothing spline. Background is the number of HFMD-associated hospitalizations by week while the light grey bars represent weeks in school vacations. (**B**)The growth rate of the estimated daily number of HFMD per day. The grey blocks represent the main HFMD epidemic period identified in each year.

Figure 1 shows the weekly HFMD-associated hospitalizations in Hong Kong between 1 January 2010 and 31 December 2014. Larger epidemics were observed in 2010 and 2013. A main summer peak from May to August was observed annually and the exponential growth phase usually started around March (Figs 1 and 2). Another milder autumn wave often occurred from September through early-October and sometimes extended into the winter. The HFMD epidemics usually peaked around the start of the summer holidays, and other school vacations usually fell outside the main peak of HFMD epidemics. Hong Kong has a humid summer with relatively stable peak absolute humidity in May and August (Fig. 1). The mean absolute humidity was 17.2 *g*/*m*^3^ and fluctuated from 12.7 to 24.5 *g*/*m*^3^ during the study periods.

The smoothed effective reproduction number (*R*_*t*_) for HFMD began to increase above 1 during early January and late March (Fig. 1). Disregarding the exceptionally high *R*_*t*_ estimates in early 2014 due to a small number of cases, the smoothed *R*_*t*_ peaked at 1.12 to 1.24 in April or May and subsequently declined to below 1 in June or July preceding the peaks of the epidemic curve. The smoothed *R*_*t*_ usually remained above 1 for around 4 to 5 months from spring to mid-summer. A rebound of *R*_*t*_ occurred between August and October in autumn, then afterwards fluctuated below 1 for most of the time during winter. In the sensitivity analyses, *R*_*t*_ peaked at 1.17 to 1.26 by assuming a mean serial interval of 2 days; while *R*_*t*_ peaked at 1.42 to 1.60 when assuming the mean as 7 days. A longer serial interval led to larger uncertainty in the *R*_*t*_ estimates and slightly shortened the duration with estimated *R*_*t*_ ≥ 1 (Supplementary Fig. 1). The assumption on case-hospitalization risk (CHR) did not affect the point estimate of *R*_*t*_ but will result in narrower 95% confidence intervals for a lower CHR (Supplementary Fig. 2).

**Figure 3:**
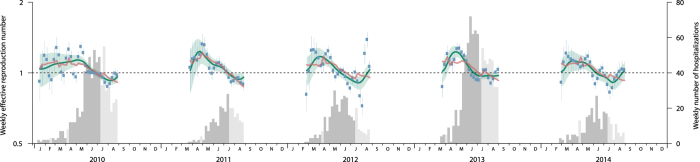
Weekly effective reproduction number (Rt) of HMFD during the study period in Hong Kong, 2010–2014. Blue dots and vertical solid line represent non-smoothed weekly *R*_*t*_ of HFMD and its 95% CI estimated from the epidemic curve during the main epidemic periods; green line and the shades represent the smoothed weekly *R*_*t*_ and its 95% CI estimated from the epidemic curve; red line represents *R*_*t*_ estimated from the model fitted without adjusting autocorrelation. The histogram is the epidemic curve of the HFMD-associated hospitalizations by week while the light grey bars represent weeks in school vacations during the main epidemic periods.

The regression model fitted with between-year effects, the depletion of susceptibles, absolute humidity and school vacations (Model 4 in Table 1, Fig. 3) explained 35% of the variance in *R*_*t*_ during the main epidemic periods. Depletion of susceptibles explained the most (19%) of the variance observed in *R*_*t*_ while between-year effects explained 13%. Absolute humidity and school vacations explained 2% and 1% of the variance in *R*_*t*_ respectively, though our results suggest absolute humidity does not explain the variance after accounting for autocorrelations (Table 2). For the sensitivity analysis which accounted for autocorrelations, the total variance in *R*_*t*_ explained was 26% (Table 2).

We found a negative association between the depletion of susceptibles and *R*_*t*_ of HFMD (coefficients ranging from −0.14 to −0.03 among different years) and a marginally insignificant positive association between absolute humidity and the reproduction number (0.11, 95% confidence interval: −0.01 to 0.22) (Table 3). A significant positive association was also found between holidays and *R*_*t*_ while the association was not significant after adjusting for autocorrelation (Supplementary Table 1). Autocorrelogram and partial autocorrelogram of model residuals suggest unaccounted autocorrelations (Supplementary Fig. 3); however, results from models with or without adjustment for autocorrelation were very similar (Table 2 and Supplementary Tables 1 and 2), except that the association between holidays and *R*_*t*_ was not significant when accounted for autocorrelation. Sensitivity analyses on the end of HFMD epidemic period showed similar results (Table 2 and Supplementary Table 2). Results from models stratified by EV71 activity were also similar with our main findings (Supplementary Tables 3 and 4). We also did not find significant associations between other meteorological factors and HFMD transmission, except for temperature in one model accounting for the autocorrelation of *R*_*t*_ (results not shown).

## Discussion

HFMD epidemics in Hong Kong occurred at least once per year and larger epidemics occurred every 2 to 3 years, similar to the pattern in other subtropical and tropical regions such as Macau, Taiwan, Singapore and Vietnam^24^,^25^,^26^. The main spring-summer peak usually started around March and peaked in June or July, while a milder autumn peak occurred after the start of a new school year in September. Smaller winter epidemics, which sometimes occurred in other subtropical regions, were also observed in 2012 and 2014 in Hong Kong^1^,^2^,^27^,^28^.

The estimated *R*_*t*_ usually exceeded 1 by March each year, corresponding to the exponential growth phase of the HFMD epidemic. *R*_*t*_ peaked in around April to May, suggesting that HFMD transmission was most intense well before summer. *R*_*t*_ dropped below 1 in June or July and rebounded to above 1 around September, probably driven by a new cohort of susceptible children entering kindergartens and the increase in contact rates among children in the new school year. However, further confirmatory study is needed to demonstrate the difference in transmission between the entry versus higher grades in kindergartens. *R*_*t*_ usually fluctuated below the threshold of 1in winter although mild HFMD activity can be observed in some years, indicating a generally low HFMD transmission during winter.

The estimated annual peak values of *R*_*t*_ ranged from 1.14 to 1.24 in the study period, indicating a relatively moderate HFMD transmissibility in Hong Kong. A transmission study in mainland China using a susceptible-infected-recovered model estimated a slightly higher *R*_*t*_, ranging from 1.4 to 1.6 across different geographical regions^22^. In our study, the yearly intercepts *log*(*R*_0_*S*_0 _**_j_), which is the logarithm of the product between *R*_0_ and the fraction of susceptibles at the beginning of each year, were estimated to range from −0.19 to 0.07 between 2008 and 2014, translating to an *R*_0_ ranging from 1.7 to 2.2 assuming 50% susceptibility at the start of the epidemic^4^,^29^,^30^. Two studies using similar statistical methods estimated that the *R*_0_ of HFMD ranged from 2.5 to 5.5 for the causative enterovirus serotypes CA16, CA6 and EV71 in Hong Kong and Singapore^20^,^21^. The estimates appear to be higher than that in our study, as the studies focused on institutional outbreaks rather than at the community level. In addition, *R*_0_ was highly sensitive to the assumed incubation period (*R*_0_ reduced by up to 50% for incubation period of 3 days instead of 5 days^20^) and the incubation period was assumed to be 5 days in the two studies, which is towards the upper end of the commonly quoted incubation period^31^,^32^.

Our results indicate that 35% of the variance in weekly *R*_*t*_ for HMFD can be explained by between-year effects, the depletion of susceptibles, absolute humidity and school holidays (Table 1). The order for potential driving factors of HFMD in our main results remained the same as that in the sensitivity analysis (explaining 22–49% of the variance in *R*_*t*_), indicating that the relative importance of the above factors were not affected by the definition of the epidemic period^23^. The depletion of susceptibles appears to be the most important driving factor of HFMD transmission, while absolute humidity and school holiday seem to have limited effects on HFMD transmission.

Between-year effects also explained a noticeable portion (13%) of the variation in HFMD transmission in Hong Kong. One possible reason was the alternating predominant serotype with different basic transmissibility and associated immunity in the community^5^,^20^,^21^. For example, 71% of the HFMD outbreaks in 2013 were associated with the subtype CA6, but only less than 30% for other years in our study period^5^. In the following year, only around 10% of the outbreaks were associated with the subtype CA6, with a similar proportion as other subtypes CA4, CA10 and EV71^5^. The remaining unexplained variance in *R*_0_ may be partly due to factors such as the different control measures in the kindergartens and schools which were difficult to measure. The unexplained variance may also due to stochasticity and individual-level factors.

Our results showed an insignificant positive association between absolute humidity and *R*_*t*_. Together with the low R^2^ explained, the absolute humidity may have limited effect on the transmission of HFMD in Hong Kong. We did not identify any other important driving meteorological factors (e.g. rainfall and sunshine) on HFMD transmission, with an exception for temperature under the model adjusting for autocorrelations (data not shown). A number of studies have reported a positive association between HFMD incidence and meteorological factors such as temperature and relative humidity, although some of the studies were conducted in temperate regions^6^,^7^,^8^,^9^,^10^,^11^,^12^,^13^. Once an epidemic takes off, the number of new cases will continue increasing even when *R*_*t*_ decreases but stays above 1. This feature somehow makes our results less comparable with previous findings which mostly focused on incidence. Another possible reason for the inconsistency is that Hong Kong is a subtropical city with relatively high temperature (median 27.0 °C, IQR 21.7–28.7 °C) and relative humidity (median 83.1%, IQR 79.4–86.4%) in most of the main epidemic periods and transmission of HFMD may be most efficient at a temperature of 23.9 °C and higher relative humidity^22^. Our model is therefore underpowered to detect the full impact of the meteorological factors due to the small number of days when the transmission is not active in Hong Kong. More evidence on the underlying biological mechanism of HFMD transmission is needed to improve the understanding of the potential effect of meteorological factors.

Summer or spring school breaks with duration longer than one month were reported to be associated with lower HFMD transmission^22^. Our results however did not suggest that school holidays substantially reduced the transmission of HFMD in Hong Kong. This may be partially explained by the fact that Hong Kong has relatively short school holidays except for the summer holidays. In many cases, *R*_*t*_ has already been decreasing well before the summer holiday (Fig. 2). Besides, previous findings showed that household plays an important role in HFMD transmission, where the transmission risk among siblings could reach 84%^33^. Public playground, which younger children would go for during school holidays, was reported as a risk factor of HFMD transmission^18^,^19^. We did not attempt to examine the potential effect of reactive school closure due to HFMD outbreaks, during which the social activities of the children may have a very different pattern from those in holidays. There were large HFMD outbreaks in 2010 in Hong Kong which led to some school closures and had significantly raised the awareness and preparedness in the schooling setting in the subsequent years. Considering the high social cost and enhanced preventive measures already in place at schools, the additional effect of school closure to reduce HFMD transmission may be limited. Furthermore, even if the EV71 vaccine would be available in Hong Kong in the future, it may have limited effect on HFMD transmission at the population, as the EV71 serotype was responsible for less than 20% of the HFMD outbreaks in Hong Kong^5^. However, EV71 is disproportionately responsible for the most severe HFMD infections^1^.

This study focused on the transmissibility of HFMD which has more direct implications on the control measures, while incidence depends on the combined effect of transmissibility and time to symptom appearance. Our study has several limitations. First, our estimations were based on hospitalized HFMD cases and may not reflect trend of population incidence reliably if the CHR changed substantially within a short period. Second, *R*_*t*_ could be overestimated if there were substantial imported cases or differences in transmissibility among serotypes. Third, we adopted the dates of summer vacations from the Education Bureau, but some kindergartens started schools earlier in August. This may partly explain the rebound of *R*_*t*_ slightly earlier than the start of the school year in September for most schools. Finally, we could not establish causation between HFMD transmission and various potential driving factors. However, the lack of association between meteorological variables and HFMD transmissibility may indicate limited effectiveness in weather-based strategies, especially for places such as Hong Kong with strengthened hygiene measures in schools.

## Conclusions

Transmissibility of HFMD in Hong Kong was relatively moderate in 2010–14. Depletion of susceptibles was the most important driving factor of the HFMD transmission dynamics. Meteorological factors and school vacations appear to have limited impact on HFMD transmission in Hong Kong.

## Methods

### Data sources

The weekly numbers of HFMD-associated hospitalizations between 1 January 2010 and 31 December 2014 in all public hospitals in Hong Kong are publicly available from the Centre for Health Protection (CHP) website^34^. The year-end and mid-year populations of Hong Kong for the years 2009 through 2014 were obtained from the Census and Statistics Department of Hong Kong^35^. Weekly and daily population were estimated by linear interpolation assuming the growing rate of population were uniform during each half-year interval. Daily counts of HFMD cases (*n*_*s*_) were derived from the weekly data as follows: we first calculated the weekly HFMD-associated hospitalizations rate *I*_*t*_ by dividing the weekly number by the interpolated weekly population. The cumulative incidence of HFMD-associated hospitalizations (*C*_*t*_) in each week *t* was then calculated. We used cubic spline interpolation to obtain the cumulative daily incidence (*C*_*s*_), and took the daily difference of *C*_*s*_ to derive daily incidence (*I*_*s*_). The case-hospitalization risk (CHR), which is defined as the risk of hospitalization among all HFMD cases, was assumed to be constant at 1.3%, based on a previous study in Hong Kong^2^. Finally, *n*_*s*_was derived using daily incidence, case-hospitalization risk and daily population.

We obtained the daily mean temperature (°C) and daily relative humidity (%) between 2010 and 2014 from the Hong Kong Observatory^36^. Absolute humidity was shown to have a more direct effect on the activity of several viruses and hence was used in our main analysis^37^,^38^,^39^. We calculated absolute humidity (*g*/*m*^3^), using Bolton’s conversion formula based on mean temperature and relative humidity^40^. Weekly absolute humidity was calculated as the arithmetic mean of daily absolute humidity in the corresponding week.

School vacations considered in the study include the Christmas, New Year, Chinese Lunar New Year, Easter and school summer holidays, which usually last for at least one week for most kindergartens and schools in Hong Kong. The period of school vacations was determined according to the guideline of school calendar issued by the Education Bureau^41^. The summer holiday was defined as from mid-July to the end of August, while the other holidays were defined as the week(s) when most of the general holiday days fell.

### Effective reproduction number

We adopted the likelihood-based approach proposed by Wallinga *et al.* and Cauchemez *et al.* to estimate the daily reproduction number (*R*_*s*_)^42^,^43^ accounting for right censoring, which is outlined below. We first calculated the relative probability (*p*_*ks*_) that cases with illness onset on day *k* were infected by cases showing symptoms on day *s*, based on an assumed serial interval distribution *w*(·). We then obtained *X*_*s*_, the number of secondary case infected by cases on day *s*, by assuming a binomial distribution Bin(*n*_*s*_, *p*_*ks*_) and observable infected cases governed by *w*(·). *R*_*s*_ was calculated by dividing *X*_*s*_ with *n*_*s*_. The weekly *R*_*t*_ was calculated as the geometric mean of the estimated daily *R*_*s*_. We assumed a negligible number of imported cases in the study period. The serial interval of HFMD was assumed to follow a Weibull distribution with mean 3.7 days and standard deviation 2.6 days^33^. Sensitivity analyses were conducted assuming serial intervals with mean 2 days and 7 days respectively and a CHR of 0.6% and 2.8%. To capture the overall trend of HFMD transmissibility in Hong Kong, we also estimated the smoothed *R*_*t*_ based on spline smoothed average weekly incidence with the lowest generalized cross-validation (GCV) score^44^.

### Potential factors of HFMD transmissibility

To further examine the potential factors that influence HFMD transmission, we used the linear regression approach as proposed by te Beest *et al.*^23^. Weekly *R*_*t*_ was used as proxy of HFMD transmission. *R*_*t*_ was assumed to be proportional to the basic reproduction number *R*_0_, scaled by the between-year effects, depletion of susceptibles and effects of absolute humidity (*AH*) and school vacations (*V*). Based on the above assumption, the linear regression model can be expressed as^23^:


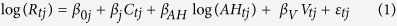


where *R*_*tj*_ is the effective reproduction number in week *t* of year *j*; *β*_*oj*_ denotes the between-year intercepts, which represents different susceptibility in the beginning of each year *j*; *C*_*tj*_ is the weekly cumulative incidence of HFMD up to week *t-1*. The coefficients *β*_*j*_, *β*_*AH*_ and *β*_*V*_ represent the effect of the yearly depletion of susceptibles, absolute humidity and school vacations. To assess the potential impact of autocorrelation, we conducted a sensitivity analysis by fitting models accounting for autocorrelations in *R*_*t*_.

The analysis was restricted to the main wave of HFMD epidemics, defined as the period from the beginning of exponential growth phase through the last week of August for each year. The exponential growth phase was defined as the period when the estimated daily number of HFMD increased steadily and the growth rate (Δ*n*_*s*_) continuously increased for no less than two months at the beginning of an epidemic. This allowed for the different start time of HFMD epidemic in each year (Fig. 2) and controlled for the new cohort of students admitted to the kindergartens in September each year. We also carried out sensitivity analyses by defining the end of HFMD epidemic period in July, September or October. Other meteorological factors such as temperature, relative humidity and air pressure were also tested. We also carried out a sensitivity analysis by stratifying the years of EV71 activity to account for the potential impacts of changing predominant serotypes.

All analyses were conducted in R version 3.1.1 (R Foundation for Statistical Computing, Vienna, Austria). We provided detailed statistical methods in the web appendix.

## Additional Information

**How to cite this article**: Yang, B. *et al.* Transmission of Hand, Foot and Mouth Disease and Its Potential Driving Factors in Hong Kong. *Sci. Rep.* **6**, 27500; doi: 10.1038/srep27500 (2016).

## Supplementary Material

Supplementary Information

## Figures and Tables

**Table 1:** Variance explained by factors of HFMD transmission in Hong Kong without adjusting autocorrelation, 2010–14.

Model	Regression Terms	Driving factors added to the model	*R*^2^	Δ*R*^2^	df	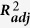
Model 1	*β*_1_*C*_*ij*_	Depletion of susceptibles	0.19	0.19	132	0.18
Model 2	*β*_0*j*_ + *β*_*j*_*C*_*ij*_	Between-year effects	0.32	0.13	124	0.27
Model 3	*Model* 2 + *β*_*AH*_ log (*AH*_*ij*_)	Absolute humidity	0.33	0.01	123	0.27
Model 4	*Model* 3 + *β*_*V*_*V*_*ij*_	School holidays^a^	0.35	0.02	122	0.29

^a^School holidays include the New Year, Chinese New Year, Easter, summer holiday and Christmas holidays.

**Table 2:** Variance Explained by Factors of HFMD Transmission in Hong Kong Using Different Ends of Study Period, 2010–14.

Driving factors	End by Jul		End by Aug		End by Sep		End by Oct	
	Model A^a^	Model B^b^	Model A	Model B	Model A	Model B	Model A	Model B
Depletion of susceptibles	0.24	0.21	0.19	0.15	0.17	0.15	0.20	0.16
Between-year effects	0.19	0.14	0.13	0.10	0.07	0.06	0.09	0.05
Absolute humidity	0.05	0.00	0.01	0.00	0.00	0.01	0.00	0.01
Holidays	0.01	0.00	0.02	0.01	0.01	0.00	0.00	0.00
Total R^2^	0.49	0.35	0.35	0.26	0.25	0.22	0.29	0.22

^a^Model A refers to model without considering autocorrelation.

^b^To account for autocorrelation, Model B used the residuals of the effective reproduction number 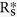, given by the 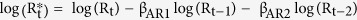.

**Table 3:** Regression Estimates of Factors Associated With HFMD Transmission in Hong Kong, 2010–14.

Driving factors	Coefficient^a^	95% CI
Yearly intercept		
2010	−0.19	(−0.50, 0.12)
2011	0.07	(−0.01, 0.16)
2012	0.01	(−0.07, 0.08)
2013	0.01	(−0.07, 0.09)
2014	0.01	(−0.07, 0.09)
Yearly depletion of susceptibles^b^^,^^c^		
2010	−0.04	(−0.06, −0.02)***
2011	−0.14	(−0.20, −0.08)***
2012	−0.07	(−0.11, −0.03)***
2013	−0.03	(−0.05, −0.02)***
2014	−0.11	(−0.16, −0.05)***
Holiday		
No	ref	
Yes	0.06	(0.00, 0.12)*
Absolute humidity (g/m^3^)	0.11	(−0.01, 0.22)

*P < 0.05; **P < 0.01; ***P < 0.001.

^a^The estimated additive effect on the model dependent variable R_t_.

^b^In the linear regression model, the coefficients for yearly intercept and yearly depletion of susceptibles both are compounds of the fraction of susceptibles at beginning of each year (E_0j_), so there are in total five pairs of coefficients for yearly intercept and yearly depletion of susceptibles^23^.

^c^Variable of cumulative incidence is in scale of 10^−6^.
