# Supplementary material for: Transmission of Hand, Foot and Mouth Disease and Its Potential Driving Factors in Hong Kong
Source: Sci Rep. 2016 Jun 7;6:27500. doi: 10.1038/srep27500 (PMC4895171; doi:10.1038/srep27500)

Transmission of Hand, Foot and Mouth Disease and Its Potential Driving Factors in Hong Kong, 2010-2014

Bingyi Yang[1], Eric H. Y. Lau[1*], Peng Wu[1], Benjamin J. Cowling[1]

[1] WHO Collaborating Centre for Infectious Disease Epidemiology and Control, School of Public Health, Li Ka Shing Faculty of Medicine, The University of Hong Kong, Hong Kong Special Administrative Region, China.

*Corresponding author: ehylau@hku.hk

Supplementary information

Statistical methods 2
Estimating effective reproduction number 2
Statistical framework 2
Serial interval 2
Estimate daily count of HFMD cases 3
Distribution of R_t 4
Identification of the main epidemic periods 5
Measurement of absolute humidity 5
Linear regression model 6
Summary of the model 6
Other meteorological factors 7
Sensitivity analysis 8
Main epidemic periods 8
EV71 activity 8
Serial interval 9
Case-hospitalization risk (CHR) 9
References 10
Supplementary Tables 12
Supplementary Table 1. 12
Supplementary Table 2. 13
Supplementary Table 3. 14
Supplementary Table 4. 15
Supplementary Figures 17
Supplementary Figure 1. 17
Supplementary Figure 2. 18
Supplementary Figure 3. 19

Statistical methods

Estimating effective reproduction number

Statistical framework

We estimated the daily effective reproduction number R_s following the likelihood-based approach proposed by Cauchemez et al. [1], which is an extension of Wallinga and Teunis's method [2]. n_s denotes the number of hand, foot and mouth (HFMD) cases showing symptoms on day s and X_s is the number of infectees of the n_s cases. R_s is then calculated as $X_s \,/\, n_s$ according to the definition [3]. It is rare to observe X_s directly, though it can be inferred from the observed epidemic curve and the serial interval distribution [2]. By considering the potential transmission networks as described by Wallinga et al. and Cauchemez et al. [1,2], the relative probability that cases with illness onset on day k have been infected by cases with illness onset on day s is

$$p_{ks} = \frac{(n_s - 1\{k = s\})w(k - s)}{\sum_{l \le S}(n_l - 1\{k = l\})w(k - l)}$$

where $w(.)$ is the probability mass function of the serial interval distribution. In theory, a primary case and its infectee can show symptoms on the same day but a case cannot be infected by itself. Therefore, an indicator function $1\{\}$ was used to avoid counting the case itself as a potential primary case.

Serial interval

With limited studies on the serial interval of HFMD, we assumed the mean serial interval as 3.7 days (standard deviation 2.6 days) from the only available transmission study of enterovirus 71 in Taiwan [4]. We assumed a Weibull distribution for the serial interval as the distribution is not available from available studies. Different values and distributions of serial interval were used in the sensitivity analysis.

Estimate daily count of HFMD cases

According to a previous study, the case-hospitalization risk (CHR) of HFMD cases in Hong Kong ranged from 0.6% to 2.8% with an average of 1.3% between 2001 and 2009 [5]. We therefore assumed the CHR as a constant of 1.3% during our study period. Within the study period, weekly and daily population P_t and P_s were estimated by performing linear interpolation.

The daily incidence was first smoothed to capture the main trend of the reproduction number in Figure 1. In the main analysis, we derived the weekly cumulative incidence of hospitalizations (C_t) up to week *t* according to the relation:

$$C_t = \frac{\sum_1^t H_j}{P_t}$$

where H_t is the cumulative sum of weekly count of hospitalization and P_t is the weekly population in week *t*. Then we used cubic spline interpolation [6] to obtain the cumulative daily incidence of hospitalizations C_s. The daily incidence of hospitalizations I_s on day *s* was calculated by $C_s - C_{s-1}$.

We derived the average incidence of hospitalizations I_t in week t according to the relation:

$$I_t = \frac{H_t}{P_t \times 7}$$

Cubic smoothing spline interpolation was used to obtain the daily incidence I_s by minimizing the generalized cross validation score. The daily number of HFMD n_s on day *s* was calculated by $n_s = I_s P_s / CHR$.

Distribution of R_t

Based on Cauchemaz's method [1], we first estimated X_s which denotes the number of secondary cases infected by cases showing symptoms on day *s*. Considering the issue of right censoring, X_s can be decomposed into cases showing symptoms on or before day S, $X_s^-(S)$, and cases showing symptoms after day S, $X_s^+(S)$. $X_s^-(S)$ follows a sum of Binomial distributions:

$$X_s^-(S) \sim \sum_{k \leq S} Bin(n_{k,}\, p_{ks})$$

The expectation and variance of $X_s^-(S)$ therefore can be obtained by the followings:

$$E(X_s^-(S)) = \sum_{k \leq S} n_k\, p_{ks}$$

$$Var(X_s^-(S)) = \sum_{k \leq S} n_k\, p_{ks}(1 - p_{ks})$$

We assumed $X_s \sim Poisson(n_s l_s)$, where l was given a a vague prior distribution $Gamma(10^{-5},\ 10^{-5})$. $X_s^+(S)$ will then follow a Negative Binomial distribution:

$$X_s^+(S) \sim NB(X_s^-(S) + \alpha;\ \frac{n_s W_{sS} + \beta}{n_s + \beta})$$

where W_{sS} is the cumulative probability that the generation interval is equal to or shorter than $S - s$.

Under the assumption that the l has a vague prior, Cauchemez [1] derived an approximation for the expectation and variance of $X_s(S)$:

$$E(X_s(S)) \approx \frac{E(X_s^-(S))}{W_{sS}}$$

$$Var(X_s(S)) \approx \frac{Var(X_s^-(S))}{(W_{sS})^2} + \frac{1 - W_{sS}}{(W_{sS})^2}\, E(X_s^-(S))$$

Eventually, we obtained daily reproduction number R_s simply according to the relation $R_s = X_s / n_s$. The weekly reproduction number R_t was calculated as the geometric mean of R_s within the corresponding week. The variance of $\log(R_t)$ was calculated using the following approximation by delta-method:

$$Var(R_t) \approx \left(\frac{R_t}{7}\right)^2 \sum_k \frac{Var(R_k)}{R_k^2}$$

where R_k refers to the daily effective reproduction number for day *k*.

Identification of the main epidemic periods

We identified the main epidemic periods for each year starting from the exponential growth phase and ended by the last week of August (Figure 2). The exponential growth phase was defined as the period when the estimated daily number of HFMD kept growing and the growth rate (Δn_s) continuously increased for no less than two months at the beginning of an epidemic according to the basic feature of epidemics [7].

Measurement of absolute humidity

Absolute humidity (AH) reflects the actual content of water vapor in the air at a given temperature and was expressed as g/m^3 in the study. We derived daily AH from relative humidity (RH, in percentage) and mean temperature (F, in Celsius) according to the following equation:

$$AH = c \times \frac{P_s(F) \times RH}{(F + 273.1) \times 100}$$

where c is a consistant of 2.166824 $gK/$J, which is approximately calculated from the molecular weight of water vapor over the gas content of water vapor. $P_s(F)$ refers to the saturation vapor pressure in Pa given temperature F and was calculated as [8]:

$$P_s(F) = 611.2 \times e^{\frac{17.67 \times F}{F + 243.5}}$$

Weekly absolute humidity was calculated as the arithmetic mean of daily AH within the corresponding week.

Linear regression model

Summary of the model

We used a linear regression model proposed by te Beest et al [9] to explore the correlation between HFMD transmission and potential driving factors. Weekly effective reproduction number R_t reflected HFMD transmissibility which depends on the potential factors including the depletion of susceptibles (E), absolute humidity (AH) and school vacations (V). We assumed R_t is a function of the basic reproduction number R_0 according to the relation:

$$R_t = R_0 E_t AH_t^{\beta_{AH}} e^{\beta_V V_t}$$

where β_{AH} and β_V are the parameters of effects of absolute humidity and school vacations. After taking the between-year effect into account, the equation of linear regression was derived as [9]:

$$\log(R_{tj}) = \beta_{oj} + \beta_j C_{tj} + \beta_{AH} \log(AH_{tj}) + \beta_V V_{tj} + \varepsilon_{tj}$$

where R_{tj} is the weekly effective reproduction number in week t of year j; β_{0j} is the intercept and equals to $\log(R_0 E_{0j})$ (E_{0j} refers to the proportion of susceptibles in the beginning of year j); β_j is the coefficients for the yearly depletion of susceptibles, which equals to -c_j/E_{0j} (c_j is a constant determined by the cumulative incidence of each year j); C_{tj} is the cumulative incidence of HFMD cases up to week t-1 of year j (in scale of per 1 million); AH_{tj} is the weekly mean absolute humidity; and V_{tj} is a binary variable indicating whether the week is in school vacation or not.

We estimated the autocorrelation and partial autocorrelation of residuals from the fitted regression model without adjusting auto-correlation terms. The residuals from the model show autocorrelation (Supplementary Figure 3). We therefore fitted a model adding the autoregressive terms of reproduction numbers up to lag 2:

$$\log(R_{tj}) = \beta_{oj} + \beta_j C_{tj} + \beta_{AH} \log(AH_{tj}) + \beta_V V_{tj} + \beta_{AR1} \log(R_{(t-1)j})$$
$$+ \beta_{AR2} \log(R_{(t-2)j}) + \varepsilon_{tj}$$

where β_{AR1} and β_{AR2} are the coefficients for the auto-regressive terms. ε_{tj} is assumed to follow a normal distribution. The ACF and PACF plots did not indicate strong autocorrelation and partial autocorrelations after adjustment of autoregressive terms (Supplementary Figure 3). Results from the models with or without adjusting autocorrelation were very similar (Table 1-3, Supplementary Table 3), which suggests the autocorrelation in R_t would not affect our main findings.

Other meteorological factors

We included absolute humidity as the meteorological factor in the main analysis. Previous studies reported temperature, relative humidity and air pressure positively correlated with the incidence of HFMD [10-12]. We therefore also included the above variables in the regression model but did not find significant associations with HFMD transmission, except for temperature in one model accounting for the autocorrelation of R_t (results not shown). In fact, absolute humidity is highly correlated with temperature, relative humidity and air pressure (data not shown). We finally chose absolute humidity as it synthesizes all the above meteorological variables and the AIC and BIC scores suggested a good model fitness when including absolute humidity, comparing to model including other meteorological factors.

Sensitivity analysis

Main epidemic periods

We conducted a sensitivity analysis by defining other cutoffs for the main epidemic periods at the end of July, September and October respectively, in order to examine their potential impacts on the results. Models adjusting or without adjusting for autocorrelation were also fitted. The relative order of the potential driving factors were the same as our main results and the results did not suggest an association between absolute humidity and R_t, except for the case when the defined epidemic period ended early by the end of July (Table 3 and Supplementary Table 2). The definition of the main epidemic periods seems to have limited impacts on our main findings.

EV71 activity

EV71 was usually with higher case-hospitalization risks comparing to other serotypes in Hong Kong [5]. We therefore conducted a sensitivity analysis stratifying the study periods by EV71 activities. We defined the EV71 activity by comparing the proportion HFMD outbreaks attributed to EV71 in outbreaks associated with CA6, CA16 or EV71.
We defined the year 2010 and 2013 as lower EV71 activity years when EV71 was responsible for 4.4% and 11.3% of HFMD outbreaks while the year of 2011, 2012 and 2014 as the higher EV71 activity (accounting for 20.7% to 36.1% of the outbreaks) [13]. Models adjusting or without adjusting for autocorrelation were fitted. The relative importance of the potential driving factors were similar with our main results (Supplementary Table 3). No significant association between R_t, and absolute humidity was founded except for the case in years with lower EV71 activity and without adjusting autocorrelation (Supplementary Table 4). EV71 activity seems to have no impacts on our main findings.

Serial interval

A sensitivity analysis was also conducted assuming a serial interval with mean 7 days and 2 days respectively with a standard deviation of 2.6 days. Results from the sensitivity analysis indicate that a longer serial interval of HFMD would lead to larger estimated R_t in the growth phase of the spring-summer epidemics (Supplementary Figure 1). The maximum values of R_t during the exponential growth phase of spring-summer waves were estimated to be 1.17 to 1.26 in the study period. Assuming a mean serial interval of 2 days; while R_t peaked at 1.42 to 1.60 when assuming the mean as 7 days (Supplementary Figure 1). A longer serial interval led to larger uncertainty in the R_t estimates and slightly shortened the duration with estimated $R_t \geq 1$ (Supplementary Figure 1). We also estimated R_t by assuming a Gamma or Lognormal distribution for the serial interval and found similar results as those assuming a Weibull distribution. Results were similar to our main analysis when $R_t$were estimated assuming different mean serial intervals.

Case-hospitalization risk (CHR)

We also estimated R_t assuming a CHR of 0.6% and 2.8% respectively throughout the study period. Results indicate that the assumption on CHR would not affect the point estimate of R_t and will result in narrower 95% confidence intervals for a lower CHR (Supplementary Figure 2). Assuming a stable CHR, data on hospitalizations provided consistent estimates of R_t, given the lack of outpatient information. However it may be challenging if the CHR changed substantially in a short period [14].

References

1 Cauchemez, S. *et al.* Real-time estimates in early detection of SARS. *Emerg Infect Dis* **12**, 110-113 (2006).

2 Wallinga, J. & Teunis, P. Different epidemic curves for severe acute respiratory syndrome reveal similar impacts of control measures. *Am J Epidemiol* **160**, 509-516 (2004).

3 Cowling, B. J., Ho, L. M. & Leung, G. M. Effectiveness of control measures during the SARS epidemic in Beijing: a comparison of the Rt curve and the epidemic curve. *Epidemiol Infect* **136**, 562-566 (2008).

4 Chang, L. Y. *et al.* Transmission and clinical features of enterovirus 71 infections in household contacts in Taiwan. *JAMA* **291**, 222-227 (2004).

5 Ma, E., Lam, T., Chan, K. C., Wong, C. & Chuang, S. K. Changing epidemiology of hand, foot, and mouth disease in Hong Kong, 2001-2009. *Jpn J Infect Dis* **63**, 422-426 (2010).

6 Hastie, T. J. & Tibshirani, R. J. *Generalized additive models*. Vol. 43 (CRC Press, 1990).

7 Ferguson, N. M. *et al.* Planning for smallpox outbreaks. *Nature* **425**, 681-685 (2003).

8 National Oceanic and Atmospheric Administration. *Relative humidity and dewpoint temperature from temperature and wet-bulb temperature*. Available at: http://www.srh.noaa.gov/images/epz/wxcalc/rhTdFromWetBulb.pdf. (Accessed: 8 2015)

9 te Beest, D. E., van Boven, M., Hooiveld, M., van den Dool, C. & Wallinga, J. Driving factors of influenza transmission in the Netherlands. *Am J Epidemiol* **178**, 1469-1477 (2013).

10 Ma, E., Lam, T., Wong, C. & Chuang, S. K. Is hand, foot and mouth disease associated with meteorological parameters? *Epidemiol Infect* **138**, 1779-1788 (2010).

11 Huang, Y. *et al.* Effect of meteorological variables on the incidence of hand, foot, and mouth disease in children: a time-series analysis in Guangzhou, China. *BMC Infect Dis* **13**, 134 (2013).

12 Wei, J. *et al.* The effect of meteorological variables on the transmission of hand, foot and mouth disease in four major cities of shanxi province, China: a time series data analysis (2009-2013). *PLoS Negl Trop Dis* **9**, e0003572 (2015).

13 Centre for Health Protection. *Update on Situation of Enterovirus Infection.* (2015) Available at: http://www.chp.gov.hk/files/pdf/update_on_situation_of_ev_infection.pdf. (Accessed: December 13 2015)

14 Cowling, B. J. *et al.* The effective reproduction number of pandemic influenza: prospective estimation. *Epidemiology* **21**, 842-846 (2010).

Supplementary Tables

Supplementary Table 1. Regression estimates of factors of HFMD transmission in Hong Kong adjusted by autocorrelation, 2010-14.

Factors	Coefficient	95% CI
**Yearly intercept**		
2010	-0.08	(-0.27, 0.11)
2011	0.04	(-0.01, 0.09)
2012	0.01	(-0.04, 0.05)
2013	0.00	(-0.04, 0.05)
2014	0.00	(-0.05, 0.04)
**Yearly depletion of susceptibles**†		
2010	-0.03	(-0.04, -0.02) ***
2011	-0.09	(-0.13, -0.06) ***
2012	-0.06	(-0.08, -0.03) ***
2013	-0.02	(-0.03, -0.01) ***
2014	-0.07	(-0.11, -0.04) ***
**Holiday**		
No	ref	
Yes	0.02	(-0.01, 0.06)
**Absolute humidity**	0.06	(-0.01, 0.13)
**R_{AR1}**	0.86	(0.74, 0.98) ***
**R_{AR2}**	-0.67	(-0.79, -0.55) ***

† Variable of cumulative incidence is in scale of 10-6.

** P < 0.05; ** P < 0.01; *** P < 0.001*

R^2 of the model is 0.77.

Supplementary Table 2. Regression estimates of factors of HFMD transmission in Hong Kong using different ends of study period, 2010-14

Factors	End by Jul		End by Sep		End by Oct	
	Model A*	Model B†	Model A	Model B	Model A	Model B
**Yearly intercepts**						
2010	**-0.36 (-0.64, -0.07)**	-0.14 (-0.31, 0.02)	0.16 (-0.12, 0.44)	0.14 (-0.04, 0.31)	0.10 (-0.12, 0.32)	0.11 (-0.02, 0.25)
2011	0.07 (0.00, 0.15)	0.03 (-0.01, 0.08)	0.05 (-0.03, 0.14)	0.03 (-0.02, 0.08)	0.04 (-0.03, 0.12)	0.02 (-0.03, 0.07)
2012	0.02 (-0.05, 0.09)	0.02 (-0.02, 0.05)	0.00 (-0.08, 0.08)	0.00 (-0.05, 0.05)	0.01 (-0.07, 0.08)	0.00 (-0.05, 0.04)
2013	0.00 (-0.08, 0.08)	0.01 (-0.04, 0.05)	0.04 (-0.04, 0.12)	0.02 (-0.03, 0.08)	0.03 (-0.05, 0.11)	0.02 (-0.03, 0.07)
2014	0.02 (-0.05, 0.10)	0.00 (-0.03, 0.04)	0.01 (-0.08, 0.09)	-0.01 (-0.06, 0.04)	0.00 (-0.07, 0.08)	-0.01 (-0.05, 0.04)
**Yearly depletion of Susceptibles**						
2010	**-0.05 (-0.08, -0.03)**	**-0.04 (-0.05, -0.02)**	**-0.01 (-0.03, 0.00)**	**-0.01 (-0.02, 0.00)**	**-0.02 (-0.03, -0.01)**	**-0.01 (-0.02, -0.01)**
2011	**-0.17 (-0.25, -0.10)**	**-0.10 (-0.14, -0.06)**	**-0.05 (-0.09, -0.01)**	**-0.04 (-0.06, -0.01)**	**-0.05 (-0.08, -0.02)**	**-0.03 (-0.05, -0.02)**
2012	**-0.11 (-0.15, -0.07)**	**-0.08 (-0.10, -0.05)**	-0.02 (-0.05, 0.01)	**-0.02 (-0.04, 0.00)**	**-0.03 (-0.05, -0.01)**	**-0.02 (-0.03, -0.01)**
2013	**-0.04 (-0.06, -0.02)**	**-0.03 (-0.04, -0.02)**	**-0.02 (-0.03, -0.01)**	**-0.01 (-0.02, -0.01)**	**-0.02 (-0.03, -0.01)**	**-0.01 (-0.02, -0.01)**
2014	**-0.16 (-0.22, -0.10)**	**-0.10 (-0.13, -0.06)**	-0.04 (-0.08, 0.00)	**-0.03 (-0.05, 0.00)**	**-0.04 (-0.07, -0.01)**	**-0.03 (-0.05, -0.01)**
**Holiday**						
No	ref	ref	ref	ref	ref	Ref
Yes	0.03 (-0.03, 0.08)	0.01 (-0.02, 0.03)	-0.03 (-0.07, 0.01)	-0.02 (-0.05, 0.00)	-0.02 (-0.06, 0.02)	-0.02 (-0.05, 0.00)
**Absolute humidity**	**0.17 (0.07, 0.28)**	**0.09 (0.02, 0.15)**	-0.03 (-0.13, 0.08)	-0.02 (-0.09, 0.04)	0.00 (-0.08, 0.08)	-0.01 (-0.06, 0.04)
**R_{AR1}**	NA	**0.87 (0.75, 0.98)**	NA	**0.88 (0.76, 1.00)**	NA	**0.89 (0.78, 0.99)**
**R_{AR2}**	NA	**-0.64 (-0.75, -0.54)**	NA	**-0.63 (-0.75, -0.51)**	NA	**-0.63 (-0.74, -0.52)**
**R^2 (%)**	48.7	85.3	24.6	71.5	29.3	73.7

** Model A refers to model without considering autocorrelation while † Model B refers to model adjusting autocorrelation.*

Estimates in bold format indicate statistical significant results with p-value < 0.05.

Supplementary Table 3. Variance explained by factors of HFMD transmission in Hong Kong stratified by EV71 activity, 2010-14

Driving factors	Lower EV71 activity[a]		Higher EV71 activity[b]	
	Model A*	Model B†	Model A	Model B
**Susceptibles depletion**	0.27	0.25	0.27	0.23
**Between-year effects**	0.03	0.02	0.05	0.01
**Absolute humidity**	0.05	0.00	0.00	0.01
**Holidays**	0.03	0.00	0.02	0.01
**Total R^2**	0.38	0.27	0.34	0.22

a Years with lower EV71 includes 2010 and 2013 (EV71 accounted for 11.3% and 4.4% of the outbreaks).

b Years with higher EV71 includes 2011, 2012 and 2014 (EV71 accounted for 20.7-36.4% of the outbreaks).

** Model A refers to model without considering autocorrelation.*

† Model B used outcome variable R_s^*, which was modified by autocorrelation.

Supplementary Table 4. Regression estimates of factors associated with HFMD transmission in Hong Kong stratified prevalence of EV71, 2010-14.

**Driving factors**	**Years with lower EV71[a]**		**Years with higher EV71[b]**	
	**Model A[c]**	**Model B[d]**	**Model A**	**Model B**
**Yearly intercept[a]**				
2010	**-0.46 (-0.89, -0.02)**	-0.05 (-0.33, 0.23)	NA	NA
2011	NA	NA	0.11 (-0.34, 0.56)	-0.08 (-0.38, 0.22)
2012	NA	NA	-0.07 (-0.17, 0.03)	-0.03 (-0.09, 0.03)
2013	-0.01 (-0.09, -0.07)	0.01 (-0.04, 0.05)	NA	NA
2014	NA	NA	-0.07 (-0.16, 0.03)	-0.04 (-0.10, 0.02)
**Yearly depletion of susceptibles[†§]**				
2010	**-0.05 (-0.08, -0.03)**	**-0.03 (-0.04, -0.01)**	NA	NA
2011	NA	NA	**-0.12 (-0.19, -0.06)**	**-0.10 (-0.15, -0.05)**
2012	NA	NA	**-0.06 (-0.10, -0.01)**	**-0.06 (-0.10, -0.03)**
2013	**-0.04 (-0.06, -0.02)**	**-0.02 (-0.03, -0.01)**	NA	NA
2014	NA	NA	**-0.09 (-0.15, -0.02)**	**-0.08 (-0.13, -0.04)**
**Holiday**				
No	ref	ref	ref	ref
Yes	**0.08 (-0.01, 0.16)**	0.00 (-0.05, 0.06)	0.05 (-0.03, 0.13)	0.04 (-0.01, 0.09)
**Absolute humidity (g/m^3)**	**0.21 (0.05, 0.37)**	0.05 (-0.05, 0.16)	0.03 (-0.13, 0.19)	0.08 (-0.03, 0.19)
**R_{AR1}**	NA	**0.80 (0.62, 0.99)**	NA	**0.90 (0.73, 1.07)**
**R_{AR2}**	NA	**-0.64 (-0.81, -0.46)**	NA	**-0.72 (-0.91, -0.53)**
**R^2**	0.38	0.27	0.34	0.22

a Years with lower EV71 includes 2010 and 2013 (EV71 accounted for 11.3% and 4.4% of the outbreaks).

b Years with higher EV71 includes 2011, 2012 and 2014 (EV71 accounted for 20.7-36.4% of the outbreaks).

c Model A refers to model without considering autocorrelation.

d Model B used outcome variable R_S^*, which was modified by autocorrelation.

§ In the linear regression model, the coefficients for yearly intercept and yearly depletion of susceptibles both are compounds of the fraction of susceptibles at beginning of each year (E_{0j}), so there are in total five pairs of coefficients for yearly intercept and yearly depletion of susceptibles [23].

† Variable of cumulative incidence is in scale of 10-6.

Supplementary Figures

Supplementary Figure 1. Sensitivity analysis on length of serial interval. A: Weekly number of hospitalized HFMD cases in Hong Kong from 1 Jan 2010 to 31 Dec 2014. B, C and D: Estimated daily R_t with 95% confidence interval assuming serial interval with mean 3.7, 2 and 7 days respectively. The standard deviation was assumed to be 2.6 days for all situations. The dotted lines represent the threshold of R_t=1.

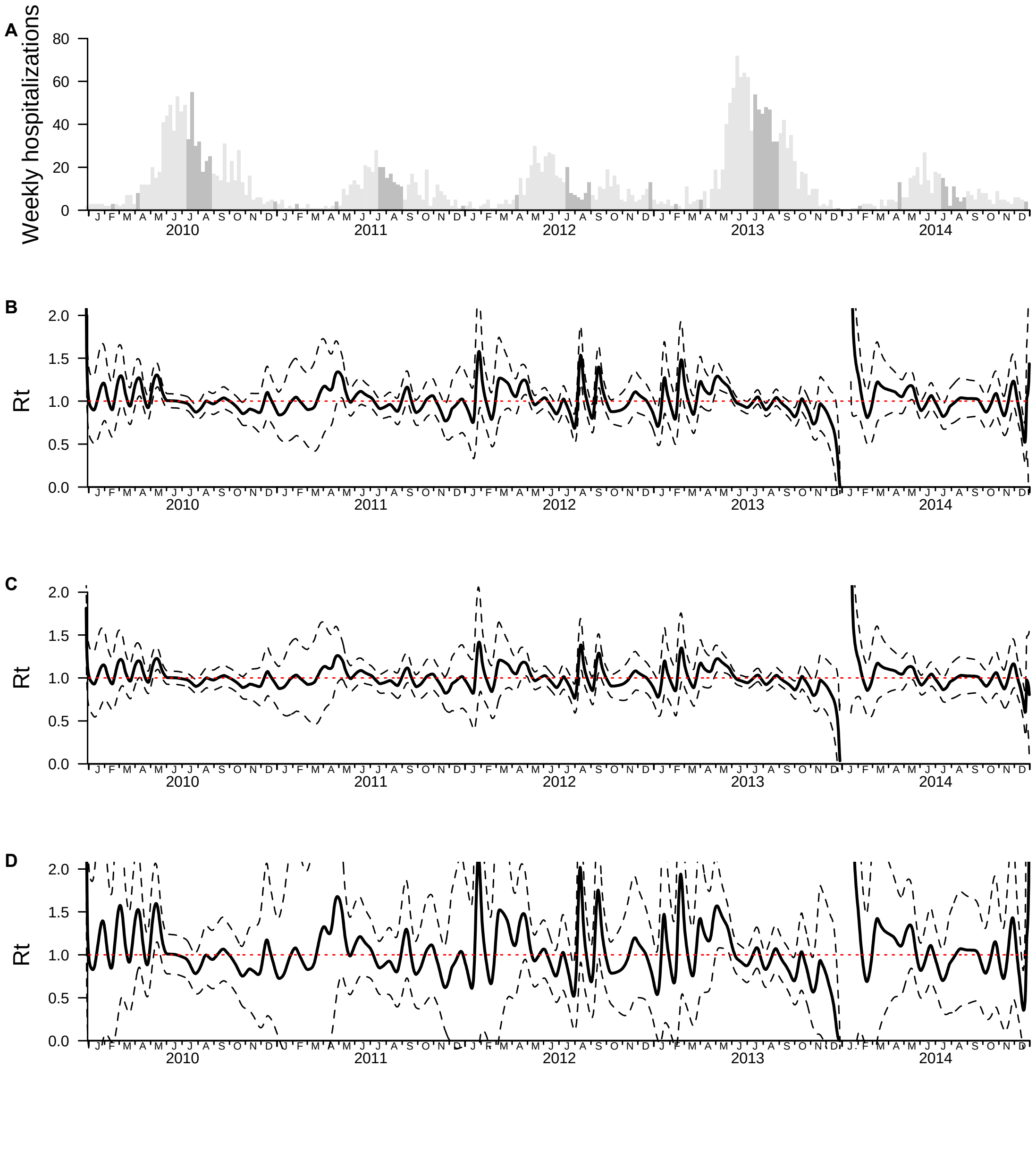

Supplementary Figure 2. Sensitivity analysis on case-hospitalization risk (CHR). A: Weekly number of hospitalized HFMD cases in Hong Kong from 1 Jan 2010 to 31 Dec 2014. B, C and D: Estimated daily R_t with 95% confidence interval assuming CHR as 1.3%, 1.6% and 2.8% respectively. The dotted lines represent the threshold of R_t=1.

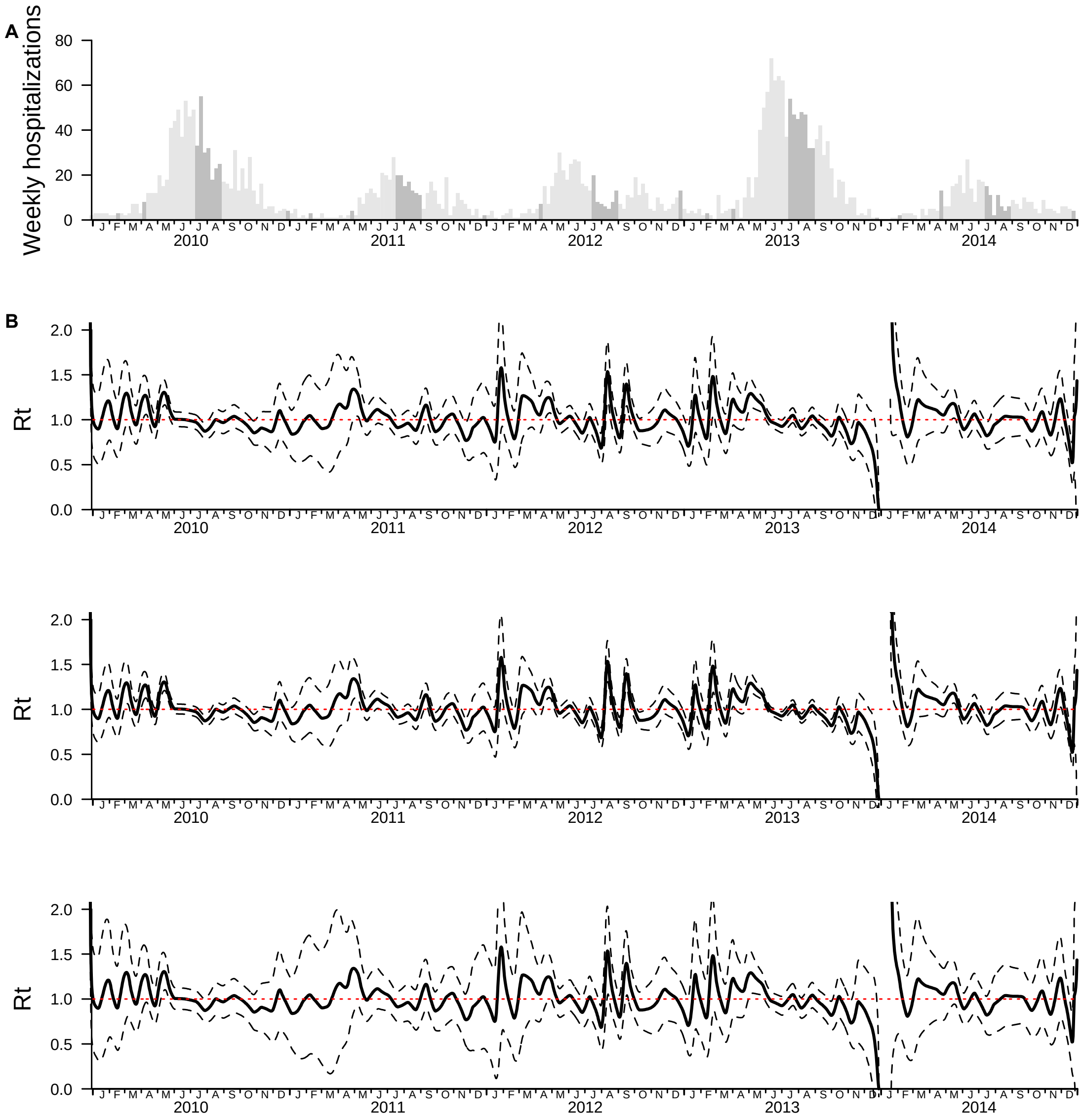

Supplementary Figure 3. ACF and PACF of residuals of model fitted with or without adjusting autocorrelation. Panel A and B, model without adjusting autocorrelation. Panel C and D, model after adjusting autocorrelation. The dashed lines represent the bounds of statistical significance.

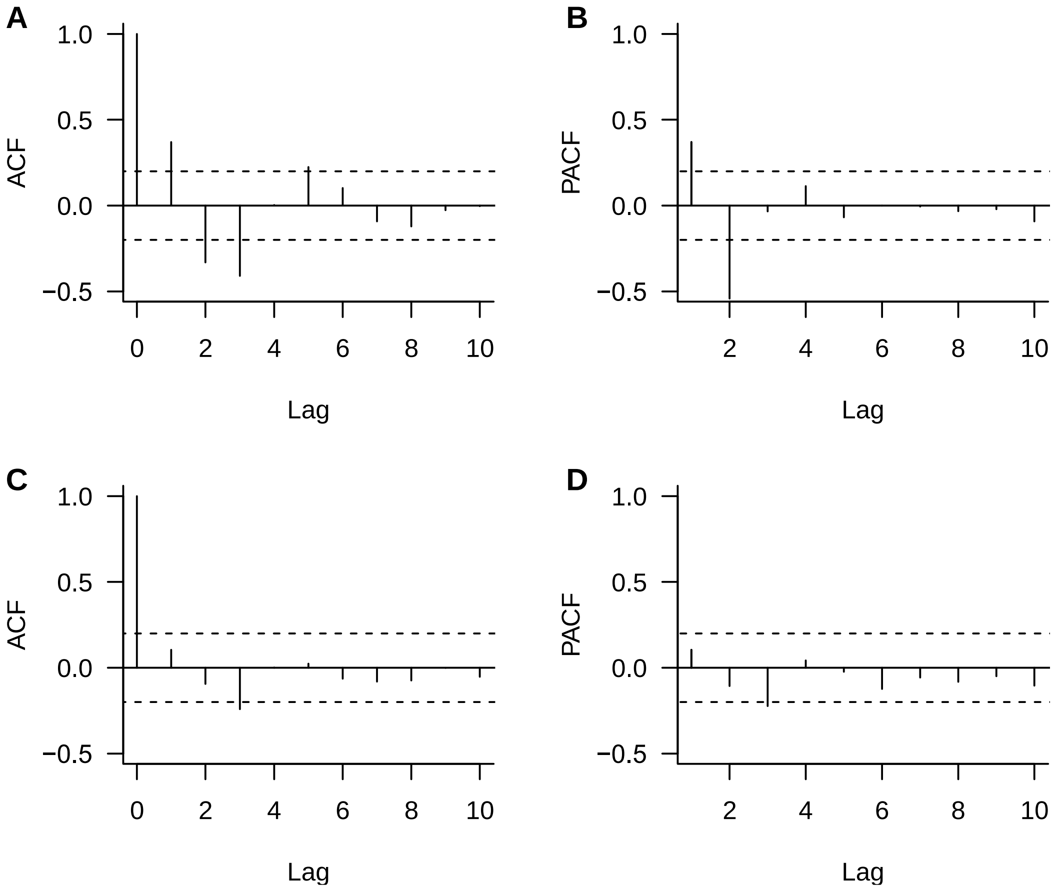

Supplement: Supplementary Information [file srep27500-s1.pdf]
